# The use of 6-0 glycomer 631 for perineal urethrostomy in male cats: 314 cases (2013–2023)

**DOI:** 10.3389/fvets.2025.1515477

**Published:** 2025-04-28

**Authors:** Kimery Hankins, Laurie Zacher-Coy

**Affiliations:** ^1^Central Texas Veterinary Specialty & Emergency Hospital, Austin, TX, United States; ^2^University of Missouri College of Veterinary Medicine, Columbia, MO, United States

**Keywords:** cats, perineal urethrostomy (PU), 6-0, glycomer 631, complications

## Abstract

**Objective:**

To assess the prevalence of postoperative complications in cats that underwent perineal urethrostomy (PU) in which 6-0 glycomer 631 was used to suture urethral mucosa to skin.

**Animals:**

314 male cats.

**Procedures:**

Medical records for cats that received a PU at Central Texas Veterinary Specialty & Emergency Hospital between 2013 and 2023 were assessed. Details including signalment, clinical status, additional surgical procedures, and postoperative complications were recorded.

**Results:**

18 of 314 (5.7%) cats developed minor complications, and 7 of 314 (2.2%) cats developed major complications that required surgical revision or resulted in humane euthanasia.

**Clinical relevance:**

Using 6-0 glycomer 631 suture is acceptable for apposition of urethral mucosa to skin in cats that undergo PU. Minor complication rates were lower and percentage of cats requiring revision surgery comparable to values reported in previous studies in which absorbable, nonabsorbable, or larger suture types were used.

## Introduction

Perineal urethrostomy (PU) is performed in male cats to alleviate distal urethral obstruction, especially recurrent obstructions that have failed previous medical management. Feline idiopathic cystitis (FIC, previously known as feline lower urinary tract disease), urethrolithiasis, trauma to the distal urethra, and idiopathic obstructions are a few conditions that can indicate the need for PU (1–4). The surgical technique involves suturing the pelvic urethral mucosa to the skin in the perineal region and therefore completely circumventing the narrow diameter of the penile urethra (5).

Previous studies have assessed various suture types to appose the urethral mucosa to the skin. Nonabsorbable suture material has been used due to its inert properties and minimal tissue reactivity (1, 6). However, complication rates are similar for cats having PU with absorbable suture material compared to nonabsorbable suture (7, 8). At one institution, there was no difference in complications between 4–0 nylon versus 4–0 or 5–0 polydioxanone in cats undergoing PU; there were no major complications in either group (7).

Glycomer 631 is uncommonly chosen for PU closure. To the authors’ knowledge, no published studies have evaluated the use of this suture type and size and subsequent complication rates for PU. In addition, while suture sizes of 6-0 are reported in urogenital surgeries (9), no studies have documented its use in PU. The primary objective of this study was to assess postoperative complication rates in cats following PU in which 6-0 glycomer 631 was used for urethral mucosa to skin apposition. The authors hypothesized that glycomer 631 would be associated with complication rates comparable to rates previously reported for other suture materials and sizes used for PU.

## Materials and methods

Electronic medical records from the Central Texas Veterinary Specialty & Emergency Hospital were searched for billing triggers for perineal urethrostomy to identify all cats that underwent PU from January 2013 through December 2023. Cats were included in the study if PU had been performed, the medical record was complete and available for review, and any postoperative follow-up evaluation had been conducted. Cats that did not have 6-0 glycomer 631 utilized for closure or did not have at least 2 weeks of available follow-up were excluded. Any data collected after 2 weeks was defined as long-term follow-up. Cats that required additional surgical procedures during the same anesthetic episode were eligible for inclusion in the study.

The information collected from the medical records of cats at the time of surgery consisted of signalment, age, body weight, reason for urethral obstruction if known, the presence of a perioperative urinary tract infection (UTI) based on urinalysis or culture results, additional surgical procedures performed, and surgeon comments regarding visible or palpable trauma to the urethra at the time of surgery if applicable. All procedures were performed by a board-certified veterinary surgeon.

### Surgical technique

Completion of PU in all cats followed the traditional principles as outlined by Wilson and Harrison (5). All cats were placed in either dorsal recumbency with the legs pulled forward toward the shoulders or sternal recumbency with pelvic limbs extended off the end of the surgical table and elevated in Trendelenburg fashion.

In all cases, an elliptical incision around the base of the scrotum was performed, the penis isolated, and tendinous attachments of the paired ischiocavernosus muscles transected to mobilize the urethra with the penis reflected dorsally. In this position, the ventral penile ligament was also broken down using a combination of sharp and blunt dissection. The penis was then reflected laterally and ventrally to section remaining muscular attachments. Proximal dissection of the dorsal urethra was accomplished to the level of the bulbourethral glands and the retractor penis muscle sharply transected with the penis reflected ventrally. Incision of the urethra was performed with a #15 blade distally and extended proximally using tenotomy scissors. The opening of the urethra was assessed to be adequate if an 8Fr or 10Fr red rubber catheter could be easily inserted and passed to the urinary bladder. The urethral mucosa was then spatulated and sutured to skin using 6-0 glycomer 631 on a taper cut swaged-on needle with simple interrupted sutures placed dorsally at the 11:00, 12:00, and 1:00 o’clock positions. A continuous line of 6-0 glycomer 631 was then used to suture each side of the drain board. Closure of the remaining urethrostomy site was then performed based on surgeon preference. In most cases, either 3–0 or 4–0 polydioxanone was used to suture subcutaneous tissue in a simple continuous pattern followed by an intradermal pattern. Occasionally, 4–0 nylon or 4–0 polypropylene was used to close the skin in a simple interrupted or cruciate pattern necessitating suture removal at a later date.

Prior to removal of the previously placed red rubber catheter, the urinary bladder was drained of urine and flushed with sterile saline until the fluid drained was clear. Cats were then recovered with one or two Elizabethan collars in place. Standard postoperative care for all cats included providing only shredded paper litter and close monitoring of the surgical wound until discharge. Administration of medications to cats in hospital prior to discharge was at the discretion of attending surgeons and included a combination of transmucosal buprenorphine at 0.01 mg/kg every 8 h, oral prazosin at 0.5 mg/kg every 12 h, oral gabapentin at 10 mg/kg every 8 to 12 h, and oral robenacoxib at 1 mg/kg.

### Follow-up evaluation

Follow-up information was obtained through recheck examinations performed at Central Texas Veterinary Specialty & Emergency Hospital or by medical records obtained from the referring veterinarian. Complications were assessed via physical exam and recorded. Complications were defined as any adverse event that occurred that required treatment outside of the normal postoperative care (10). Sequelae, any event inherent and inevitable to the surgical procedure as previously defined by the Clavien–Dindo system (11), were not included as complications. Postoperative complications were further classified as those that occurred following anesthetic recovery as noted at any time within the follow-up period. Complications for each cat were then graded as minor or major. Minor complications included those that resolved with medical management alone or without treatment; major complications were defined as those requiring surgical intervention or cases that resulted in humane euthanasia.

### Data analysis

Minor and major postoperative complications that were assessed from medical records were collected, and descriptive analysis of information was performed. No analyses to assess normality of data were performed.

## Results

The electronic medical record search identified 468 cats that underwent PU during the study period. However, 154 cats were excluded because suture types other than glycomer 631 were utilized or incomplete medical records were assessed. The remaining 314 cats, all male, were included in the study.

The most common breeds of the 314 cats included in the study consisted of domestic shorthair (*n* = 223), domestic longhair (*n* = 24), domestic medium hair (*n* = 18), Siamese (*n* = 8), and Maine Coon (*n* = 6). Median age was 5 years (range, 0.5 to 16.0 years), and median body weight was 6 kg (range, 3.1 to 10.45 kg). Urethral obstruction was the primary complaint for all 314 cats, with FIC most common (*n* = 292 [93.0%]), followed by urethral calculi (*n* = 22 [7.0%]).

Intraoperative culture results were available for 110 cats with 33 of the 110 cultures positive for growth (30.0%). Nine specific pathogens were isolated with the most common being *Escherichia coli* (*n* = 9) and *Enterococcus* (*n* = 7). Two cats had positive growth for both *Escherichia coli* and *Enterococcus* simultaneously. Only two cats had comments recorded regarding urethral trauma intraoperatively. One cat had a urethral stricture proximally that underwent revision surgery, and the other cat had a urethral tear that was managed with an indwelling foley catheter postoperatively. The cat with the urethral tear following resolution had no additional complications recorded 2 years later. Concurrent surgical procedures were performed in 32/314 cats (10.2%). Surgical procedures included cystotomy in 22 cats and castration in 10 cats. Twelve cats had a PU performed in dorsal recumbency while the remaining cases were in sternal recumbency for the procedure.

Overall complications are summarized in Tables 1 and 2. A total of 158 cats had short-term follow-up (≤ 2 weeks) and 156 cats had long-term follow-up (> 2 weeks). Median follow-up time was 0.5 months (range 0.5 to 120 months). Eighteen out of 314 cats (5.7%) developed minor complications over the entire study period including surgical site infection (*n* = 5), urethral trauma (*n* = 4), urinary catheter placement to resolve reobstruction due to urethral spasms (*n* = 4), hemorrhage requiring blood transfusion (*n* = 2), dehiscence (*n* = 1), incisional irritation (*n* = 1), and adverse suture reaction (*n* = 1). Eleven out of 158 cats (7.0%) had minor complications in the short-term while 7/156 cats (4.5%) had minor complications in the long-term interval. Major complications that required surgical revision of the PU site were identified in 7/314 cats (2.2%) over the entire study period including six that required revision due to stricture of the original PU site. Stricture was identified at two (*n* = 2), four (*n* = 2), five, and 36 weeks following initial PU. The remaining cat that underwent surgical revision in this population was related to dehiscence of the original PU closure 2 weeks postoperatively. Three out of 158 cats (1.9%) had major complications recorded within the short-term while 4/156 cats had major complications recorded in the long-term period (2.6%). Five out of the seven cats (71.4%) underwent successful revision surgery by either shortening the urethra or removing part of the caudal ischium to allow placement of sutures more proximally in part of the urethra not previously handled. The remaining two cats were euthanized during revision surgery due to concerns regarding anatomical inability to create an appropriately sized stoma and owner decline for an alternative surgical procedure. Both cats had stricture of their previous stoma 2 and 4 weeks following initial correction. Following successful revision, none of the cats had additional complications reported after a median of 5 months (range 0.5 to 19 months). No issues following initial PU were recorded for 143 cats. Clinical signs designated as sequela were observed in 146 cats with 36 of 146 (24.7%) demonstrating continued signs of FIC, including intermittent episodes of stranguria and hematuria. Forty-four out of 146 cats (30.1%) were presumptively treated for a UTI by their primary veterinarian with no record of urine cultures available.

**Table 1:** Minor complications in 18 of 314 cats that had perineal urethrostomy using 6-0 glycomer 631 suture.

Minor complications	Number of cats	Short-term (≤2 weeks)	Long-term (>2 weeks)
Surgical site infection	5 (1.6%)	3 (1.9%)	2 (1.3%)
Urethral trauma	4 (1.3%)	4 (2.5%)	0 (0%)
Urinary catheter placement to resolve reobstruction	4 (1.3%)	1 (0.6%)	3 (1.9%)
Hemorrhage requiring blood transfusion	2 (0.6%)	2 (1.3%)	0 (0%)
Dehiscence	1 (0.3%)	0 (0%)	1 (0.6%)
Incisional irritation	1 (0.3%)	1 (0.6%)	0 (0%)
Adverse suture reaction	1 (0.3%)	0 (0%)	1 (0.6%)

A total of 158 cats had short-term follow-up and 156 cats had long-term follow-up.

**Table 2:** Major complications in 7 of 314 cats that had perineal urethrostomy using 6-0 glycomer 631 suture.

Major complications	Number of cats	Short-term (≤2 weeks)	Long-term (>2 weeks)
Stricture of previous PU site	6 (1.9%)	2 (1.3%)	4 (2.6%)
Dehiscence of initial PU site	1 (0.3%)	1 (0.6%)	0 (0%)

A total of 158 cats had short-term follow-up and 156 cats had long-term follow-up.

## Discussion

The use of 6-0 glycomer 631 resulted in a low rate of postoperative complications in cats undergoing PU. From the complications, most of these were minor with seven cats in the entire population having major complications requiring additional surgical intervention. Two of these cases were euthanized intraoperatively (0.6% mortality rate). Minor and major complication rates and percentage of cats requiring revision surgery were lower than or equivalent to previous reports in which other absorbable, nonabsorbable, or larger sized suture was used (2, 7, 8, 12). In one previous study evaluating suture material type, short-term complication rates were 14% and 11% and long-term complication rates 21% and 19% for nonabsorbable and absorbable sutures, respectively (7). No cat in either group in the previous study required revision surgery for reobstruction (7).

To the authors’ knowledge, no study has assessed the use of glycomer 631, specifically 6-0, in PU. An abstract describing a modified PU technique evaluated 216 cats and subsequent complication rates that had glycomer 631 incorporated in closure. However, the size of the suture was not indicated (13). Glycomer 631 is an absorbable, synthetic monofilament suture material prepared from glycolide, dioxanone and trimethylene carbonate that has minimal tissue reactivity. This suture is degraded quickly by hydrolysis of the polymer chains and loses approximately 60% of its tensile strength within 21 days; the material is completely absorbed within 110 days (14). Degradation is accelerated when immersed in urine, especially when alkaline urine or bacteria are present. Previous studies have examined the use of various absorbable sutures in the urinary tract and their duration (15).

While previous studies have examined the loss of tensile strength of absorbable suture materials in continuous immersion of urine (15), cats that have undergone PU do not have the suture persistently saturated. Therefore, a rapidly absorbable suture type such as glycomer 631 should retain tensile strength until healing of the urethral mucosa which is generally 5–7 days (16, 17). The resorption time is beneficial as urethrostomy sites closed with an absorbable suture material do not require suture removal at a later date (1, 8). Also, the small size of 6-0 suture contributes to less tissue reactivity and less patient discomfort as demonstrated in canine abdominal suturing and an experimental study performed in rats (18, 19). Therefore, the authors believe similar results may be inferred for cats undergoing PU closure with this type and size of suture.

Urethral stricture has been a reported complication following PU in approximately 12% to 18% of cases in cats (2, 20). However, more recent studies have shown that urethral stricture rates are less than or equivalent to the rate noted in the present study (7, 8, 12). The present study demonstrated a relatively low percentage (1.9%) of cats that required revision surgery due to stricture which was nearly equivalent to a different investigation utilizing poliglecaprone 25. In that study, out of 61 cats, only one required surgical revision because of stricture (1.6%) (8). While complications following PU in cats are common, many are minor regardless of suture material and size used (7, 8). In this study, results demonstrate that 6-0 glycomer 631 can be a suitable suture material to use for PU closure, and the size of the suture may be associated with lower or similar complication rates compared to studies utilizing larger suture (7, 8).

Previous studies also demonstrate that regardless of suture material or size, cats undergoing PU can continue to have signs associated with FIC (4, 21, 22). While PU helps manage cats with urethral obstruction secondary to FIC, it does not exclude the cat from developing recurrent lower urinary signs due to the underlying etiology (4, 16, 23). In a recent study, up to 56.7% of cats that underwent PU developed subsequent FIC (12). While the present study did not report such a high percentage, appropriate management and discussion with owners prior to surgery should be a priority for suitable long-term management of these cases. Ongoing clinical signs even after undergoing PU or revision surgery may create quality of life concerns for these cats and their owners.

Urinary tract infections are also a commonly reported complication following PU as the structural anatomy of the cat is altered thus increasing probability for an ascending infection to occur (21). In a 2019 report, 22.7% of cats that underwent PU developed a UTI afterward (24). While 110/314 cats (35.0%) in the present study had intraoperative urinary cultures performed, a correlation cannot be made for long term presence of bacteriuria or UTI in these cases. In addition, not all UTI diagnosed in the present study were from urinalyses but rather presumptively made either based on cultures or clinical signs observed. However, the data provides evidence that regardless of suture material or size utilized, UTI can be a frequent complication surrounding PU surgery in cats (2, 9, 20, 25, 26).

For soft tissue surgical procedures in small animals, there is no standardized or widely accepted grading system to categorize postoperative complications. With the formulation of the Clavien-Dindo classification scheme for surgical procedures in humans, translation can be made to veterinary medicine to appropriately account for complications that previously were underestimated or unassessed. Thus, minor and major complications were assessed for this population with other expected postoperative signs designated as sequelae. Due to the application of this type of grading system, complication rates were lower for this population compared to other studies as no previous report featured sequelae as part of their complication grading scheme but rather designated all postoperative signs as either a minor or major complication. The Clavien-Dindo scheme utilized for this study, while potentially overestimating sequela compared to other complication systems available (27), allows for reliable classification and standardization for complications. Arguably, this grading scheme should be widely adopted to allow accurate comparisons between studies with the recognition that sequelae are an inherent consequence to surgical procedures. This system could prevent overinflation of the overall complication rates outlined in other studies.

Limitations of this study include its retrospective nature, the limited or inconsistent follow-up, and the inevitable variation in assessment between surgeons. Furthermore, the recording of complications and therapies instituted were subjective based on personal experience and preferences. Thus, the incidence of various complications and treatments administered could be inaccurate and therefore influence outcome for this population. Additionally, because of the retrospective nature of the study and limitations of the medical records, no direct comparison could be made between PU performed at Central Texas Veterinary Specialty & Emergency Hospital with 6-0 glycomer 631 versus other suture materials and sizes. Characterization of complications into a grading scheme also allows for subjective interpretation and therefore bias in some instances. Ultimately, a prospective study with a variety of suture material and sizes along with comparison of suture patterns utilized for PU in cats would be needed for direct outcome comparison.

## Data Availability

The raw data supporting the conclusions of this article will be made available by the authors, without undue reservation.
